# A Study on the Susceptibility to SCC of 7050 Aluminum Alloy by DCB Specimens

**DOI:** 10.3390/ma9110884

**Published:** 2016-11-01

**Authors:** Xing Qi, Jirong Jin, Chunli Dai, Wenjuan Qi, Wangzhao He, Renguo Song

**Affiliations:** 1 Jiangsu Key Laboratory of Materials Surface Science and Technology, Changzhou University, Changzhou 213164, China; qixing321@outlook.com (X.Q.); gdjinjirong@163.com (J.J.); qwjanna@163.com (W.Q.); wzhe@163.com (W.H.); 2 Jiangsu Collaborative Innovation Center of Photovolatic Science and Engineering, Changzhou University, Changzhou 213164, China; 3 Institute of Metal Research, Chinese Academy of Sciences, Shenyang 110016, China; cldai@imr.ac.cn

**Keywords:** 7050 aluminum alloy, stress corrosion cracking, DCB specimen, aging, cathodic polarization

## Abstract

The stress corrosion cracking (SCC) of different aging states for 7050 aluminum alloy in 3.5% sodium chloride aqueous solution has been studied by means of double cantilever beam (DCB) specimens, cathodic polarization, scanning electron microscope (SEM), transmission electron microscope (TEM) and time-of-flying second ion mass spectrometer (ToF-SIMS). The results showed that the susceptibility to SCC (Iscc) of 7050 aluminum alloy decreases with increasing the aging time. When a cathodic polarization potential of −1100 mV was applied to DCB specimens, the ion current intensity of hydrogen (I_H_^+^) near the crack tip and Iscc increased obviously, thus the degree of the diffusion of hydrogen into the grain boundary become more serious. The observation of microstructure indicated that the precipitates on the grain boundary become coarse and are sparsely distributed with increasing the aging time of 7050 aluminum alloy.

## 1. Introduction

7050 aluminum alloy (AA7050) belongs to high strength Al-Zn-Mg-Cu series alloy with outstanding performance of high strength, excellent welding and processing properties which make it be one of the main structural materials in aerospace and automobile industries [1,2,3]. However this type of alloy is very sensitive to stress corrosion cracking (SCC) that means corrosion cracks will be easily expanded in internal alloy with long-term usage under corrosive environment, furthermore, there would not exist any significant omen before the fracture of alloy component [4]. Therefore it is very important to research the SCC behavior of 7050 aluminum alloy. Many researchers have published numerous articles about the research subject of SCC of high strength aluminum alloy under various heat treatment methods and environments [5,6,7,8,9]. For such a long time they still have not reached a consensus about the mechanism of SCC to this alloy because of the complexity of corrosion phenomena. X.Y. Sun et al. presented in their paper that if the width of grain boundary precipitated phase is narrow, the SCC mechanism will be hydrogen induced cracking, otherwise it will be anodic dissolution [10,11]. The primary form of corrosion for high strength aluminum alloy is pitting and intergranular corrosion in 3.5% NaCl solution. In addition, the susceptibility to SCC increased when applied a proper cathodic polarization. R.G Song et al. found that when applied a cathodic polarization to 7050 Aluminum alloy, intergranular brittle fractures could be seen on fracture surface [12]. The open potential and breakdown potential of 7050 aluminum alloy decrease and the current increase at a given potential with the growing stress which accelerate the anodic dissolution.

Recently, with the development of analytic techniques, the analysis of elements to stress corrosion cracking samples plays an increasing important role in research of corrosion behavior [13]. In the present work, the SCC behavior of 7050 aluminum alloy has been studied by double cantilever beam (DCB) specimens with different aging states and cathodic polarization applied. The Tof-SIMS was used to analyze the depth profile of alloy fracture and the diffusion degree of hydrogen inside the grain boundary, so that it is possible to evaluate the effect of hydrogen during stress corrosion cracking process. Meanwhile, transmission electron microscope (TEM) was used to examine the effect of aging on microstructure of 7050 aluminum alloy.

## 2. Experiments

7050 aluminum alloy used in the present study is an alloy plate of 55 mm thickness produced by Alcoa Company. The chemical compositions are showed in Table 1. The specimens were put in a Muffle furnace with 470 °C for 2 h then quenched with cold water, after that put in an oven with 135 °C for 8 h (under-aged), 16 h (peak-aged) and 24 h (over-aged) respectively.

**Figure 1:**
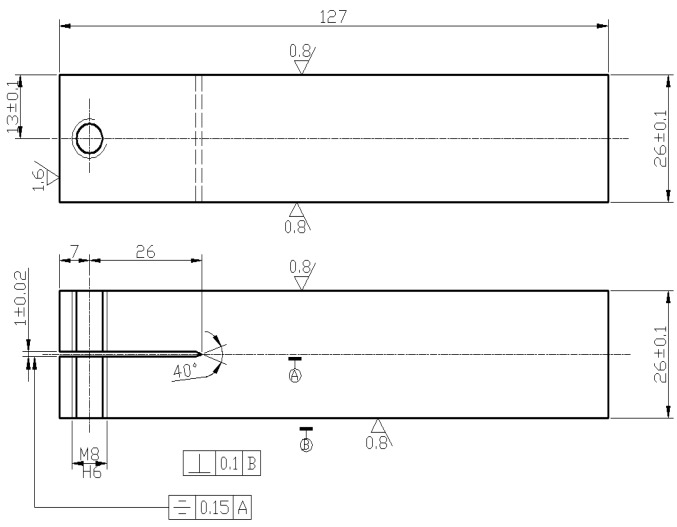
Dimensions in mm of DCB specimen.

The dimension of DCB specimen is shown in Figure 1 which designed strictly in accordance with ISO 7539. The crack expanding direction is along with the rolling direction. During the experiment, 3.5 wt % NaCl solution was used as corrosion media under 25 ± 10 °C temperature situation and HCl or NaOH was used to adjust pH value to ensure it ranged between 6.4 and 7.2.

The surface of specimens were polished successively by 1000# and 1500# sandpapers then washed with acetone and rinsed with distilled water. Every DCB specimen was pre-cracked by two M8 screws being tightened on the two sides of the specimen synchronously to generate a crack about 1–2 mm, meanwhile a reading microscope was used to measure the crack length before and after immersion in 3.5 wt % NaCl solution. A −1100 mV polarization potential was applied to DCB specimens during immersion in which a saturated calomel electrode was used as reference electrode and the platinum electrode was used as counter electrode. Meanwhile, the scan rate of the potentiodynamic test was 0.5 mV/s and the scanning rang was between −300 mV and 1200 mV. The screws were not moved during immersion thus there always a load applied on the specimen. After that, the crack lengths of all specimens were recorded every ten days.

Two 10 mm × 10 mm square specimens were produced to carry out potentiodynamic test. Only one surface of the specimen was exposed in a vessel with 500 mL 3.5% NaCl solution acted as one electrode, while other surfaces were sealed by epoxy resin. A saturated calomel electrode was used as reference electrode and the platinum electrode was used as working electrode. A potentiodynamic instrument was applied to provide power and record data.

A time-of-flying second ion mass spectrometer (ToF-SIMS 5, Muenster, Germany) was used to analyze the depth profile of crack tip on fracture surface as well collect its H^+^ intensity. Fracture morphology was examined by scanning electron microscope (SEM) (JSM-6510, Tokyo, Japan) and microstructure was observed by transmission electron microscope (TEM) (JEM-2100, Tokyo, Japan).

## 3. Results and Discussion

### 3.1. Polarization Curves of Different Aged Aluminum Alloy

**Figure 2:**
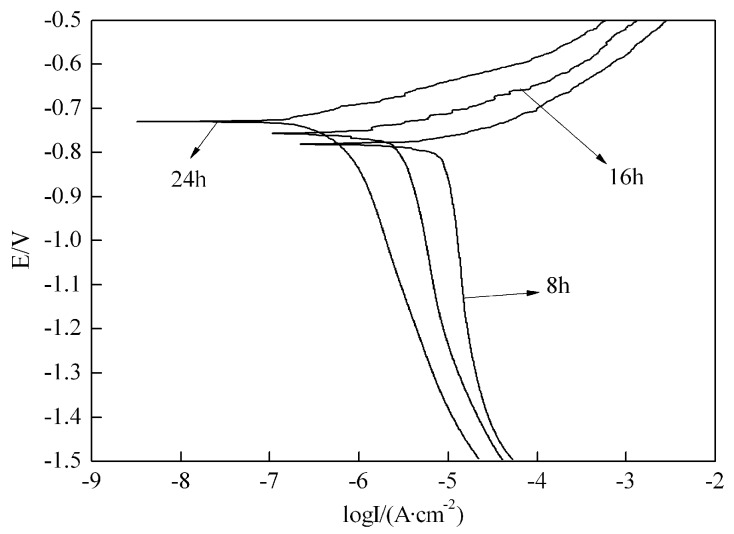
Cathodic polarization curves of 7050 aluminum alloy with different aging time.

Figure 2 shows three polarization curves of different aged 7050 aluminum alloys after immersed in 3.5 wt % NaCl solution for 24 h. The open circuit potentials of 8 h, 16 h and 24 h aged alloy were fitted out by CVIEW2 software and they were −0.7800 mV, −0.7564 mV and −0.7306 mV respectively. This indicated that the open circuit potential increased with increasing the aging time, which suggested that the corrosion resistance of 7050 aluminum alloy become better and better with increasing the aging time.

### 3.2. Effect of Aging and Cathodic Polarization on Susceptibility to SCC 

**Figure 3:**
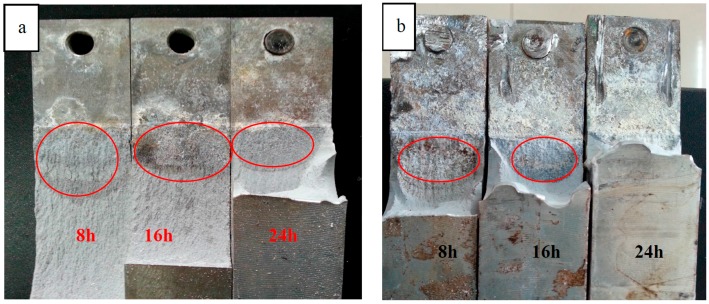
Fracture photos of AA7050 DCB specimens. (**a**) With a cathodic potential of −1100 mV; (**b**) without any cathodic potential.

Figure 3a shows the macro fracture photos of AA7050 DCB specimens immersed in 3.5 wt % NaCl solution, each applied a −1100 mV cathodic potential, the dark area (red circles) in fracture surface was the crack propagation depth which suggested that 7050 aluminum alloy with 8 h aging time had the worst corrosion resistance, and 24 h was the best. The same tendency could be seen in Figure 3b, which showed the fracture photos of AA7050 DCB specimens without any cathodic potential. The comparison between DCB specimens with and without cathodic potential indicated that the existence of a polarization potential increased the corrosion susceptibility, because we could see from Figure 3a,b that the crack propagation depth of specimens with a −1100 cathodic potential was much longer than the one without a cathodic potential under the same aging condition, especially for 24 h over aged (Figure 3b). Furthermore the crack propagation depth of the specimen immersed in 3.5 wt % NaCl solution without a cathodic potential was almost zero (24 h aged specimen in Figure 3b).

**Figure 4:**
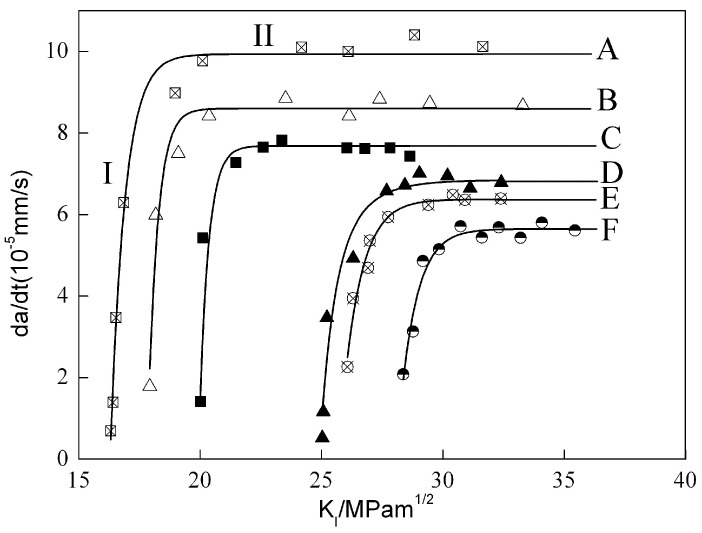
Curves of da/dt vs. K_I_ in 7050 aluminum alloy: (**A**) under aged, E = −1100 mV; (**B**) peak aged, E = −1100 mV; (**C**) under aged; (**D**) peak aged; (**E**) over aged, E = −1100 mV; (**F**) over aged.

Set a as the average crack length among under aged, peak aged and over aged alloy specimens, the crack propagation rate is determined by a–t curve, the stress intensity factor near crack tips is as follows:

(1) $$K_{I}=\frac{E\delta H{\left[3H{\left(a+0.6H\right)}^{2}+H^{3}\right]}^{1/2}}{4\left[{\left(a+0.6H\right)}^{3}+H^{2}a\right]}$$

where *δ* is the displacement on loading direction, *H* the half height length of specimens which is 13 mm, *E* Young’s modulus which changed little with different heat treatments, so it uniformly accessed to 70,560 MPa, and finally we drew out the *da/dt-K*_I_ curves of various aged specimens with or without a −1100 mV cathodic polarization potential, as showed in Figure 4. Their propagation platform rates of cracks and the critical stress intensity factors were listed in Table 2.

These curves could be divided into two regions, region I and region II. In region I, the crack growth rates of all specimens were highly depended on stress intensity factor while in region II there was no significant correlation between crack growth rate and stress intensity factor [14]. The crack growth rates are calculated by the average crack length recorded everyday through a reading microscope. The curves C, D and F in Figure 4 indicated that the crack propagation rates decreased with increasing the aging time, thus the over aged alloy DCB specimen owned the minimum propagation rate value of 5.61 × 10^−5^ mm·s^−1^, meanwhile the critical stress intensity factor increased significantly with longer aging time. This meant the stress corrosion cracking susceptibility correlated closely with aging time, longer aging time showed lower stress corrosion susceptibility. The stress corrosion resistance of AA7050 specimens with over aged treatment was much higher than the ones with under aged and peak aged treatment. The same tendency could be seen in curves A, B and E which applied the same cathodic polarization potential of −1100 mV. However, the crack growth rate of DCB specimen with cathodic potential was higher than the specimen without any cathodic potential under the same aging condition, which indicated that it was much easier to occur stress corrosion for cathodic polarized specimen than non-polarized specimen. Yang, Q. et al. [15] suggested that the reason that cathodic polarization potential would increase stress corrosion susceptibility was the generation of hydrogen under this situation. When the specimen was immersed in 3.5% NaCl solution and each applied a −1100 mV potential, more hydrogen would be generated and concentrated near crack tips which made the passive film around crack tips unstable therefore resulted in local erosion. Hydrogen atoms were liberated and absorbed when solution was transported to the crack tip and reacted with the fresh fracture surface, then it diffused to the region ahead of crack tip and the hydrogen metal interaction led to material embrittlement.

### 3.3. Effect of Aging States and Cathodic Polarization on SCC Fracture Morphology

**Figure 5:**
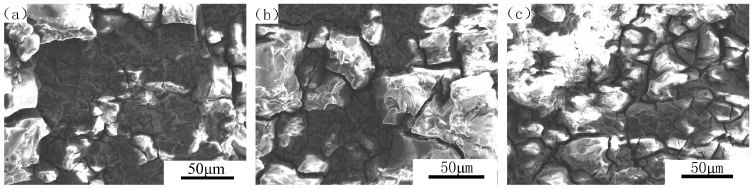
Fracture morphologies for 7050 aluminum alloy of three different aging states. (**a**) Over aged; (**b**) peak aged; (**c**) under aged.

**Figure 6:**
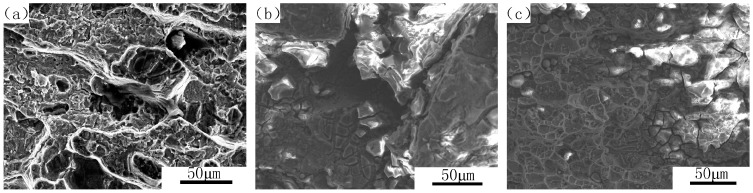
Fracture morphologies for 7050 aluminum alloy of three different aging states, E = −1100 mV. (**a**) Over aged; (**b**) peak aged; (**c**) under aged.

Figure 5 shows the SEM morphology of fracture surface of 7050 aluminum alloy with three different aging states after immersed in 3.5 wt % NaCl solution, and Figure 6 shows the SEM morphology of fracture surface of 7050 aluminum alloy with three different aging states after immersed in 3.5 wt % NaCl solution and meanwhile each applied a cathodic polarization potential of −1100 mV. It can be seen from Figure 5 and Figure 6 that the fracture type of various aged specimens was a combination of transgranular and intergranular characteristics accompanied with some local erosion. The proportion of intergranular cracks decreased with the increase of aging time. More significant embrittlement characteristic were found on 8 h aged specimens while for 24 h over aged treatment, there were more transgranular cracks. However, corrosion performed more severely compared with the ones without any cathodic potential in Figure 5 when applied a cathodic polarization potential of −1100 mV (see Figure 6). The corrosion rate of 7050 aluminum alloy was in an order of under aged specimens > peak aged specimens > over aged specimens no matter whether any cathodic polarization potential applied or not. For under aged condition, corrosion had already extended into the internal matrix, some erosion even appeared in local areas when a cathodic potential of −110 mV applied (Figure 6c). For peak aged alloy, corrosion occurred along with the grain boundary and many surface cracks could be seen on SEM morphology (Figure 5b and Figure 6b); however, for over aged alloy without a cathodic potential (Figure 5a), some dimples also appeared and many grain crystals dissolved during immersion which meant that the specimens under this state is slightly of better ductility than the others. Therefore we could concluded that over aged 7050 aluminum alloy is of the best stress corrosion resistance while under aged 7050 aluminum alloy performed the worst. Furthermore we could determine that the corrosion susceptibility was influenced by cathodic potential. When the specimen was applied a cathodic potential, the brittleness extent of fracture increased and resulted in more severely stress corrosion, which is in consistent with the results of the stress corrosion test above.

### 3.4. Effect of Aging and Cathodic Polarization on Hydrogen Distribution near Crack Tips

**Figure 7:**
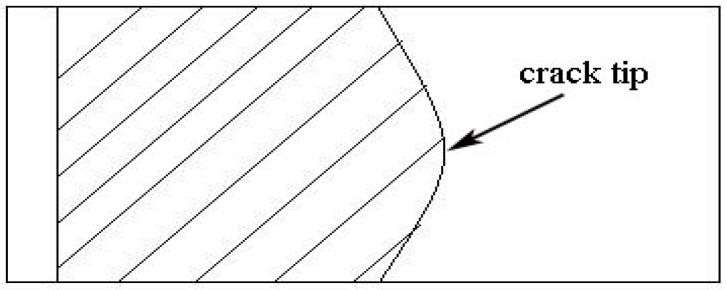
The schematic of ToF-SIMS analysis point.

**Figure 8:**
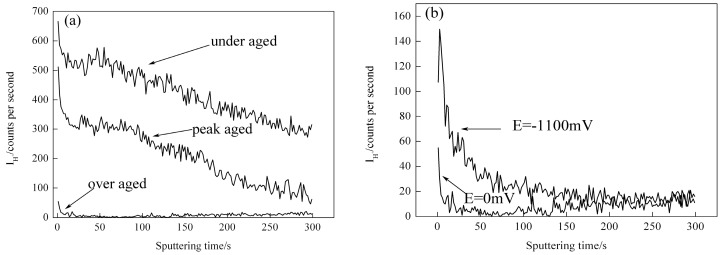
ToF-SIMS sputtering profiles for H^+^ near the crack tip. (**a**) Different aging states; (**b**) over aged state and E = −1100 mV.

The sputtering velocity of ToF-SIMS was much quicker in depth profiling. The data collection and analysis speed could be increased after every sputtering, which suggested that the trace element would not be re-oxidized during the time range when it was sputtered and collected, thus the analytical results obtained from Tof-SIMS had extremely high reliability [16]. When the crack growth rate of DCB specimens reduced to 10^−7^ mm/s, the specimens then were mechanical ruptured. The analyzed point was marked in Figure 7. Figure 8 shows the depth analysis of I_H_^+^ near crack tips, in which the vertical coordinate was the intensity of hydrogen ion flow, while horizontal coordinate meant sputtering time which was directly proportional to stripping depth on specimen surface. In the present paper, we set all the stripping depth as 300 s. Figure 8a illustrated the intensity of hydrogen ion flow of 7050 aluminum alloy with three different aging treatment, from which the I_H_^+^ near crack tips of all these specimens had a reduce tendency with the increase of sputtering time. When the sputtering time equal to 25 s, the I_H_^+^ of over aged specimen dropped to zero, and the I_H_^+^ of peak aged specimen almost dropped to zero when the sputtering time reached to 300 s. However, the I_H_^+^ was still above zero after sputtering 300 s for under aged specimen. Generally, more sputtering time meant deeper the hydrogen spread into alloy, Figure 8a indicated that the hydrogen diffusing of under aged specimens was much deeper than other two aging states which meant more hydrogen concentrated at their crack tips. Moreover the hydrogen concentration decreased with the increasing of aging time.

The grain interior structure of 7050 aluminum alloy with under aged state is mainly desoluted GP zone (segregation zone of solute atoms), these GP zones act as reversible hydrogen traps, therefore the free hydrogen atoms insider alloy will fill these traps until they are saturated which makes the hydrogen concentration near crack tips of under aged aluminum alloy much higher than peak aged and over aged states. While the precipitated phase of over aged aluminum alloy is mainly η phase particle which acts as irreversible hydrogen trap so hydrogen atoms cannot saturate in this kind of traps thus there would not be as much hydrogen concentrated near crack tips as of under aged state. In addition the precipitated phase of peak aged aluminum alloy is a mix of GP zone and η phase particle, therefore the hydrogen concentration of peak aged aluminum alloy after immersed in 3.5% NaCl solution is between the values of under aged and over aged states [17]. Figure 8b was a comparison of I_H_^+^ between aluminum alloy specimens under over aged treatment with and without a cathodic polarization potential of −1100 mV. Because hydrogen evolution reaction occurred during immersion and the new generated hydrogen atoms entered into internal matrix through diffusing which resulted in increased hydrogen concentration near crack tips and more deeply propagation towards matrix under cathodic polarization condition, some certain cathodic polarization condition would enhance hydrogen concentration near crack tips of 7050 aluminum alloy and thus increasing the stress corrosion susceptibility.

### 3.5. TEM Observations

**Figure 9:**
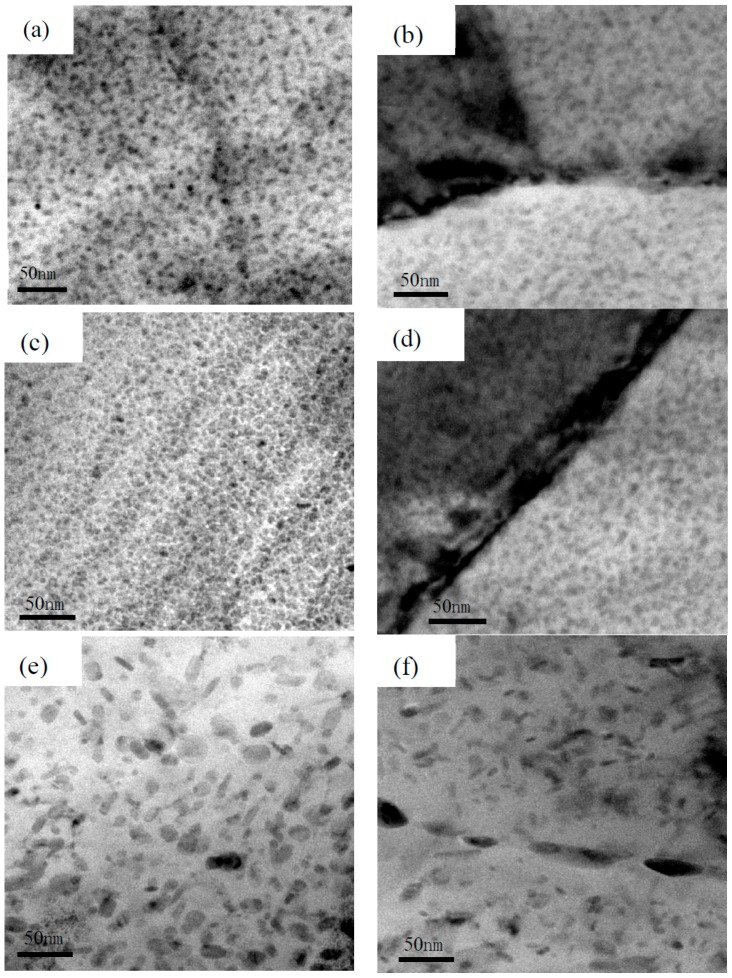
TEM bright field images of 7050 aluminum alloy with different aging states. (**a**,**b**) under aged (8 h); (**c**,**d**) peak aged (16 h); (**e**,**f**) over aged (24 h).

In order to study the effect of aging time on precipitation phases inside grain and on grain boundary, the TEM observations for under aged, peak aged and over aged 7050 aluminum alloy have been performed in this paper, as shown in Figure 9. For under aged state, the precipitated phase in grain (see Figure 9a) were small round shape characteristic with an average size of 3−5 nm, and complete and continuous grain boundary precipitates could be seen from Figure 9b, these were mainly incoherent η phase which would not dissolute under high temperature but would enrich and coarsen on grain boundary [18]. Figure 9c,d were precipitated phases inside grain and on grain boundary for peak aged aluminum alloy respectively, which were similar to under aged. However, the particle size in grain was a little bigger and the grain boundary precipitates appeared slight discontinuity compared to the under aged. In contrast, the precipitated phases inside grain and on grain boundary of over aged aluminum alloy were totally different (Figure 9e,f) from under aged and peak aged, the precipitated phases inside grain appeared strip shape mixed with some round feature, meanwhile the grain boundary precipitates looked totally discontinuous in comparison with the other two aging stages. The precipitate-free zone (PFZ) width of the three aged states showed an increase tendency with increasing the aging time, which were about 5 nm, 10 nm and 20 nm respectively.

The specimens with bigger precipitates would attract more hydrogen atoms which counted against to stress corrosion resistance (SCR), moreover, continuous chain structure of precipitated phases provided corrosion channels to develop stress corrosion crack, while completely discontinuous grain boundary precipitates would hinder the formation of anode channels thus benefit to anti stress corrosion, this is why the under aged state alloy is of the worst SCR while the over aged state is of the best SCR.

## 4. Conclusions

(1) The susceptibility to stress corrosion cracking of 7050 aluminum alloy decreased while the critical stress intensity factor K_ISCC_ increased with increasing the aging time. When applied a cathodic polarization potential of −1100 mV, the K_ISCC_ decreased significantly and aluminum alloy was much easier to be corroded.
(2) The hydrogen concentration near crack tips of fracture zone enhanced when applied a cathodic polarization potential of −1100 mV and the crack propagation moved deeper towards grain interior. In addition, the diffusion depth hydrogen into 7050 aluminum alloy matrix reduced with increasing the aging time and the hydrogen enrichment also gradually diminished.
(3) The stress corrosion cracking behavior of 7050 aluminum alloy was correlated with precipitated phases, i.e., the better stress corrosion resistance is due to small particle size and discontinuous grain boundary precipitated phases as well as wide precipitate-free zone (PFZ).

## Figures and Tables

**Table 1:** Chemical compositions of 7050 aluminum alloy.

Element	Zn	Mg	Cu	Zr	Ti	Mn	Cr	Fe	Si	Al
wt %	6.42	2.25	2.02	0.13	0.03	0.10	0.04	0.11	0.07	Balance

**Table 2:** The SCC susceptibility of 7050 aluminum alloy immersed in 3.5% NaCl solution.

Aging State	Polarization Potential (mV)	Plateau Velocity/mm·s^−1^	K_ISCC_/MPa·m^1/2^
Under aged (8 h)	/	7.64 × 10^−5^	20.02
Under aged (8 h)	−1100	9.97 × 10^−5^	16.32
Peak aged (16 h)	/	6.65 × 10^−5^	25.04
Peak aged (16 h)	1100	8.67 × 10^−5^	17.93
Over aged (24 h)	/	5.61 × 10^−5^	28.38
Over aged (24 h)	1100	6.36 × 10^−5^	26.01
